# To Ki or Not to Ki: Re-Evaluating the Use and Potentials of Ki-67 for T Cell Analysis

**DOI:** 10.3389/fimmu.2021.653974

**Published:** 2021-04-09

**Authors:** Francesca Di Rosa, Andrea Cossarizza, Adrian C. Hayday

**Affiliations:** ^1^ Institute of Molecular Biology and Pathology, National Research Council of Italy (CNR), Rome, Italy; ^2^ Department of Medical and Surgical Sciences for Children and Adults, University of Modena and Reggio Emilia, Modena, Italy; ^3^ National Institute for Cardiovascular Research, Bologna, Italy; ^4^ Immunosurveillance Laboratory, The Francis Crick Institute, London, United Kingdom; ^5^ Peter Gorer Department of Immunobiology, King’s College London, London, United Kingdom; ^6^ National Institute for Health Research (NIHR) Biomedical Research Center (BRC), Guy’s and St Thomas’ NHS Foundation Trust and King’s College London, London, United Kingdom

**Keywords:** flow cytometry, T cells, cell cycle, Ki-67, DNA dye

## Abstract

This study discusses substantive advances in T cell proliferation analysis, with the aim to provoke a re-evaluation of the generally-held view that Ki-67 is a reliable proliferation marker *per se*, and to offer a more sensitive and effective method for T cell cycle analysis, with informative examples in mouse and human settings. We summarize recent experimental work from our labs showing that, by Ki-67/DNA dual staining and refined flow cytometric methods, we were able to identify T cells in the S-G_2_/M phases of the cell-cycle in the peripheral blood (collectively termed “T Double S” for T cells in S-phase *in Sanguine*: in short “T_DS_” cells). Without our refinement, such cells may be excluded from conventional lymphocyte analyses. Specifically, we analyzed clonal expansion of antigen-specific CD8 T cells in vaccinated mice, and demonstrated the potential of T_DS_ cells to reflect immune dynamics in human blood samples from healthy donors, and patients with type 1 diabetes, infectious mononucleosis, and COVID-19. The Ki-67/DNA dual staining, or T_DS_ assay, provides a reliable approach by which human peripheral blood can be used to reflect the dynamics of human lymphocytes, rather than providing mere steady-state phenotypic snapshots. The method does not require highly sophisticated “-omics” capabilities, so it should be widely-applicable to health care in diverse settings. Furthermore, our results argue that the T_DS_ assay can provide a window on immune dynamics in extra-lymphoid tissues, a long-sought potential of peripheral blood monitoring, for example in relation to organ-specific autoimmune diseases and infections, and cancer immunotherapy.

## Introduction

Quantitation of Ki-67, a nuclear protein associated with cell cycle, is currently among the top-ranked methods to evaluate T cell proliferation, especially in human samples *ex vivo*. Readily detectable levels of Ki-67 mRNA and protein are present during the four cell cycle phases (i.e., G_1_, S, G_2_, M) and are down-regulated when cells exit cell cycle and enter into quiescence (i.e., the G_0_ phase) (1). Originally named after the first monoclonal antibody (mAb) used to identify it (2), Ki-67 protein can now be stained with a series of mAbs with different sensitivities and epitope-specificities, including some mAbs that can detect extremely low levels of the protein, even in quiescent cells (3–6). From a functional standpoint, Ki-67 supports chromosome architecture organization and nucleolar assembly upon mitosis (7, 8); helps remove cytoplasm from the reassembling nucleus during mitotic exit (9); and regulates heterochromatin compaction and gene expression in proliferating cells (10). This notwithstanding, mutant mice with disrupted Ki-67 expression are vital and fertile, grow normally, and do not show abnormalities in highly proliferative tissues, such as the intestinal epithelium (10).

Given these considerations, it is evident that the very frequent use of Ki-67 as a proliferation marker is mistaken: rather, Ki-67 discriminates between cells having detectable Ki-67 expression (Ki-67^+^) in any phase of cell cycle (i.e., G_1_, S, G_2_, M), and cells lacking it (Ki-67^-^) in the quiescent state G_0_. Notably, the G_1_ phase can be considered a cell cycle hub of highly variable duration. Thus, a Ki-67^+^ cell in G_1_ can derive either from cell cycle entry of a cell that was previously in G_0_, or a mitotic event that generates two daughter cells that sustain in G_1_. Indeed, an often-neglected notion is that the subsequent fate of a Ki-67^+^ cell in G_1_ can be any one of the following: i) to remain in G_1_ for a long time; ii) to rapidly proceed into S-G_2_/M; iii) to move into G_0_, going out of cell cycle.

Adding a layer of complexity, Ki-67 levels in G_1_ and G_0_ also depend on the time a cell has spent in that phase, as the protein is degraded continuously during G_1_ and G_0_, while it accumulates in S, G_2_, M (6). Some critical issues related to Ki-67 protein half-life emerged from a series of elegant studies addressing mouse B and T cell proliferation dynamics by *in vivo* experiments plus mathematical modeling (11–14). In some of these studies, Ki-67 staining was used in combination with other methods, including mouse treatment with bromodeoxyuridine (BrdU), a thymidine analogue which is incorporated into DNA during S-phase, and adoptive transfer of T cells labeled with carboxyfluorescein succinimidyl ester (CFSE), a fluorescent dye that is equally distributed between daughter cells upon division (11, 12). It was observed that Ki-67 level of expression by Ki-67^+^ cells may vary depending on cell division history. Thus, Ki-67 expression by CFSE-labeled T cells transferred in lymphopenic mice was extremely high in cells that had undergone many rounds of divisions, and low in recently divided cells whose proliferation was inhibited by adoptive transfer of large numbers of competing T cells (11). The possibility that Ki-67 expression could reflect a recent post-mitotic state was reinforced by BrdU pulse-chase data, showing that BrdU-labeled CD4 T_EM_ and T_CM_ cells were still Ki-67^+^ 4 days after BrdU-treatment withdrawal (12). Furthermore, it was highlighted that Ki-67 expression by a differentiated T or B cell could derive from Ki-67 protein inheritance following cell division at a previous developmental stage, rather than reflect proliferation of the Ki-67^+^ cell itself (13, 14). In short, evaluation of Ki-67 as a single marker to define a proliferative state incurs risk of misinterpretation.

In this article, we will discuss how these considerations are brought into focus by recent findings on proliferating T cells in the peripheral blood of healthy subjects and those with diseases (15, 16), including COVID-19 (17). We will briefly describe how a highly sensitive and effective flow cytometric method based on Ki-67/DNA dual staining, the T_DS_ assay, provided a practical and reliable approach to distinguish between T cells in G_1_ and those in S-G_2_/M phases of cell cycle (15–17). Finally, we will advocate incorporating the T_DS_ assay into routine immuno-monitoring.

## Ki-67/DNA Dual Staining of T Cells

Refined kinetics studies on T cell clonal expansion after vaccination in a mouse model showed that antigen-specific CD8 T cells from blood contained on average 8-fold more cells in S-G_2_/M at day 3 *versus* day 7 post-boost, even though both the percentage of Ki-67^+^ cells and the antigen-specific CD8 T cell frequency were much higher at day 7 (**Supplemental Table 1A**, and (15)). These results suggested that the peak of actively cycling cells anticipates by a few days the peak of Ki-67^+^ cells at day 7, which might reflect entry of cells into a prolonged G_1_ phase after a recent mitosis and/or cell mobilization into the blood. By contrast, only a small proportion of total CD8 T cells from control untreated mice were in G_1_, and very few were in S-G_2_/M at any time point (**Supplemental Table 1A**).

In these experiments, the limitations of using Ki-67 as a single marker were overcome by a flow cytometric method based on dual staining of Ki-67 and DNA, that allows a clear distinction of cells in G_0_ (Ki67^-^/DNA2n), from those in G_1_ (Ki67^+^/DNA2n), and from those in S-G_2_/M (Ki67^+^/2n<DNA ≤ 4n), as previously demonstrated by studies of bone marrow Hematopoietic Stem Cells (HSC) (18). When adapting this protocol to *ex vivo* analysis of antigen-specific T cells of mouse spleen, lymph nodes (LN) and blood, an unconventional strategy for data analysis was employed that included events with high Forward (FSC) and Side Scatter (SSC) (15). Such events are commonly discarded when examining lymphocytes in freshly obtained heterogeneous tissue samples, in order to exclude cell aggregates and myeloid cells that are typically more auto-fluorescent than lymphocytes.

In the mouse vaccination study, the unconventional combination of a DNA-Area/DNA-Width criterion, that is normally used in HSC cell cycle studies to exclude cell aggregates and debris (19), with a “relaxed” lymphocyte gate for FSC and SSC, allowed the ready detection of T cells in the S-G_2_/M phases of the cell cycle that might have been missed with a standard gating strategy, and offered enhanced sensitivity in measuring cells in G_1_ (15). Indeed, comparison of mouse LN data obtained applying either the conventional lymphocyte gate excluding cells with high FSC and SSC, or the “relaxed” gate that included cells with high FSC and SSC, revealed that at day 3 post-boost the conventional gate under-estimated antigen-specific CD8 T cell frequency and the Ki-67^+^ proportion among antigen-specific CD8 T cells by an average of 6-fold, and 3-fold, respectively (15). These results suggest that the current criteria of analysis of *ex vivo* mouse samples are appropriate for resting T cells, but are not optimal for activated T cells, e.g. those cycling during early phases of an immune response to vaccination.

## Validation by Image Flow Cytometry

The prospect that antigen-responding T cells proceeding into cell cycle share with blast cells traits including increasing size and modifications of internal organelles has been validated by image flow cytometry analysis of a TCR transgenic mouse CD8 T cell population following stimulation with its cognate antigen *in vitro* (16). In these studies, the combination of flow cytometry and microscopy permitted visualization and quantitative multi-parameter characterization of T cells in different phases of the cell cycle, as identified by Ki-67/DNA dual staining: thus antigen-induced T cell cycle progression corresponded to quantitative increases in SSC, nuclear size (DNA area), cell size (brightfield area), and uptake of a mitochondrial marker (16).

Notably, T cells with such features of cycling blast cells were identified in the peripheral blood of some healthy donors (HD), without any *in vitro* stimulation (16). In this context, Ki-67 staining resolved an unexpected technical issue, i.e. the presence of a few cell aggregates (about 0.3% of the CD8 T cells) that could not be eliminated based on the DNA-only criterion and which appeared as Ki-67^-^ events having 4n DNA content. Visualization of these events by image flow cytometry analysis showed that they were doublets formed by one cell sitting almost on top of another cell, thereby appearing like a shadow (“shadow” doublets) (16). This type of potential artifact involving only a tiny cell fraction had been previously reported, and solved by microscopy-based high content screening (20). The Ki-67/DNA dual staining offered the option to exclude “shadow” doublets by flow cytometry, i.e. by gating out Ki67^-^/4n DNA events (16).

## The T_DS_ Assay for Refined Immuno-Monitoring

**Figure 1:**
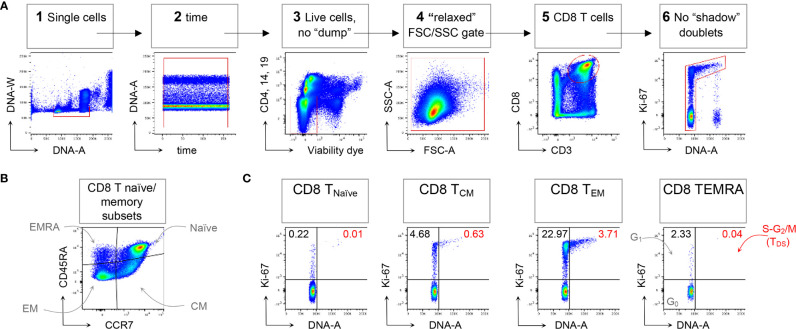
Example of CD8 T cell naïve/memory subset analysis by T_DS_ assay. HD PBMCs were stained with the viability dye eFluor 780 (eF780), the DNA dye Hoechst-33342, and fluorochrome conjugated mAbs against surface markers and Ki-67, as described (16). An example of flow cytometry analysis is shown. **(A)** Gating of viable single CD8 T cells in 6 steps: 1) DNA-A/-W singlets . Single cells having 2n≤ DNA content ≤4n were selected on the DNA-area (A) versus (vs) DNA-width (W) plot; 2) Time exclusion . Stable acquisition over time (seconds) was monitored on the time vs DNA-A plot and any events collected in case of pressure fluctuations were excluded; 3) Viable cells, no “dump” . Cells expressing CD4, CD14 and CD19, and dead cells were excluded; 4) FSC-A/SSC-A “relaxed” gate . A “relaxed” gate was used on the FSC-A vs SSC-A plot, to include highly activated and cycling lymphocytes (15); 5) CD8 T cells . CD8 T cells were gated on the CD3 versus CD8 plot; 6) Refined singlets. A few remaining doublets composed by one cell sitting on top of another (so-called shadow doublets) were excluded as Ki-67^int^/^-^ events having > 2n DNA content (16). This gating strategy was used as a base for the subsequent gates. **(B)** The following naïve/memory subsets of CD8 T cells were identified: CD45RA^+^ CCR7^+^ Naïve, CD45RA^-^ CCR7^+^ central memory (CM), CD45RA^-^ CCR7^-^ effector memory (EM), and CD45RA^+^ CCR7^-^ (EMRA). **(C)** Cell cycle phases of each naïve/memory CD8 T cell subset were defined on DNA-A vs Ki67-A plot as follows: cells in G_0_ were identified as DNA 2n/Ki67^-^ (bottom left quadrant); cells in G_1_ as DNA 2n/Ki67^+^ (upper left quadrant); cells in S-G_2_/M (or T_DS_ cells) as DNA>2n/Ki67^+^ (top right quadrant). Unpublished data in relation to (16).

Following the technical resolution of “shadow” doublets, we could consistently apply flow cytometry to detect and quantitate in HD blood T cells in the S-G_2_/M phases of cell cycle, collectively called “T Double S” for T cells in S-phase *in Sanguine*, in short T_DS_ (16). Indeed, T_DS_ were clearly evident in T_reg_ cells Fraction II (CD4^+^ FoxP3^high^ CD45RA^-^ T cells) from HD, being on average 0.82%, and suggesting ongoing immune regulation. T_DS_ were rare among conventional T cell memory subsets, and more frequently represented in CD4 and CD8 T_EM_ cells. In contrast, T_DS_ were almost completely absent among *γ*δ T cells (16). A typical example of flow cytometry data demonstrating T_DS_ enrichment in CD8 T_EM_ cells is represented in **Figure 1**, that shows general gating strategy (panel A), identification of naïve/memory CD8 T cell subsets (panel B), and cell cycle analysis for each subset (panel C).

These results prompted further investigation in human diseases employing Ki-67/DNA dual staining and flow cytometric analysis: the T_DS_ assay. In Infectious Mononucleosis (IM), the clinical manifestation of primary EBV infection, CD8 T cells specific for a single EBV immunodominant epitope contained up to 80% of cells in G_1_ and up to 20% of T_DS_ cells, whereas corresponding cells in healthy EBV carriers contained about 5% of cells in G_1_ and 0% T_DS_. In fact, T_DS_ performed better than the frequency of EBV-specific CD8 T cells for discriminating IM patients from healthy EBV carriers (16).

In Type 1 Diabetes (T1D), the T_DS_ assay was applied to CD8 T cells specific for antigens of Islets of Langerhans, the target organ of the pathogenic autoimmune attack. An evident difference in T_DS_ representation emerged between patients and HD, that was quantitated as follows: considering 0.248% T_DS_ (i.e. HD mean + 3xSD) as a threshold, individuals with T_DS_ percentage values above this $\left(T_{DS}^{+}\right)$ comprised about half of the T1D cohort, and were absent among HD (16). The two subsets of T1D patients — $T_{DS}^{+}$ and $T_{DS}^{-}$ — showed significantly different T_DS_ representation among islet-specific CD8 T cells but not among anti-viral CD8 T cells (16) or total CD8 T cells (**Supplemental Table 1B**). A prominent rise in islet-specific CD8 T_DS_ cells (>3%) was associated with an aggressive effector phenotype of the islet-specific CD8 T cells in the blood. Thus, T_DS_ measurement may have immediate clinical utility offering extra insight into the progression of a disease which can be challenging to track by other means (16).

Finally, the T_DS_ assay was used in an immune monitoring study (“COVID-IP”) of hospital-treated COVID-19 patients (17). Among the key traits of a consensus COVID-19 immune signature identified by the study was a dysregulated T cell response characterized by concurrent cytopenia, activation, proliferation, and exhaustion. The T_DS_ assay documented that patients with a severe disease progression had a higher percentage of cells in G_1_ and of T_DS_ cells among CD4 and CD8 T_EM_ cells, as compared with HD. *γ*δ T cells in G_1_ were similarly increased but those cells were not associated with increases of cells in S-G_2_/M. All such changes were less evident in patients with a moderate disease evolution, with a significant difference between the two patient groups (**Supplemental Table 1C**). Hence, in this setting too, the ready quantitation of T cells in G_1_ and T_DS_ cells could contribute to patient discrimination. Lastly, blood T cell analysis by Ki-67/DNA dual staining helped to identify critical immunological traits of COVID-19 patients with either solid or haematological cancers versustheir non-COVID counterparts, further confirming the great utility of the T_DS_ assay in immunomonitoring (21).

## Concluding Remarks

In sum, the value of Ki-67 as an informative marker of cell cycle status can be greatly enhanced by its use in combination with a DNA stain, as described in the T_DS_ assay. The routine flow cytometry application of this might usefully be used to better understand T cell biology, to monitor responses to vaccination and treatment, and to gain early warnings of spontaneous disease exacerbation or remission. The possibility of using more sophisticated approaches, based upon multilaser excitation of molecules such as CSFE for counting cell divisions, or fluorochrome-conjugated antibodies recognizing cyclins differentially expressed during the cell cycle (22) or even mass cytometry (23) may soon permit a better understanding of, and hence better deployment of, Ki-67 in tracking and studying cell cycling as a key component of immune regulation.

## Author Contributions

FD wrote the manuscript with help by AH and insightful inputs by AC. Experimental studies were performed in the labs of FD and AH. All authors contributed to the article and approved the submitted version.

## Funding

Funding for this study was provided by Royal Society grant IES\R3\170319 (to AH and FD) and Italian Minister of Research and University (MIUR) grant PRIN 2017K55HLC (to FD).

## Conflict of Interest

AH is a board member and equity holder in ImmunoQure, AG., and Gamma Delta Therapeutics, and is an equity holder in Adaptate Biotherapeutics.

The remaining authors declare that the research was conducted in the absence of any commercial or financial relationships that could be construed as a potential conflict of interest.
