# Corrigendum: To Ki or Not to Ki: Re-Evaluating the Use and Potentials of Ki-67 for T Cell Analysis

**DOI:** 10.3389/fimmu.2021.756641

**Published:** 2021-09-28

**Authors:** Francesca Di Rosa, Andrea Cossarizza, Adrian C. Hayday

**Affiliations:** ^1^ Institute of Molecular Biology and Pathology, National Research Council of Italy (CNR), Rome, Italy; ^2^ Department of Medical and Surgical Sciences for Children and Adults, University of Modena and Reggio Emilia, Modena, Italy; ^3^ National Institute for Cardiovascular Research, Bologna, Italy; ^4^ Immunosurveillance Laboratory, The Francis Crick Institute, London, United Kingdom; ^5^ Peter Gorer Department of Immunobiology, King’s College London, London, United Kingdom; ^6^ National Institute for Health Research (NIHR) Biomedical Research Center (BRC), Guy’s and St Thomas’ NHS Foundation Trust and King’s College London, London, United Kingdom

**Keywords:** flow cytometry, T cells, cell cycle, Ki-67, DNA dye

In the original article, there was a mistake in the legend for **Figure 1** as published. On the Viable cells, no “dump” gate, “CD16” was written instead of “CD19”. The correct legend appears below.

**Figure 1:**
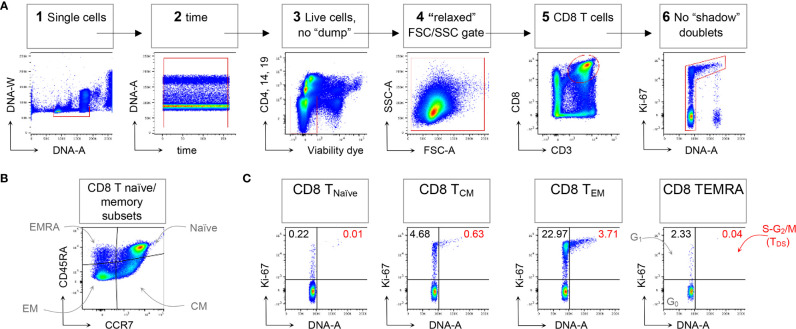
Example of CD8 T cell naïve/memory subset analysis by T_DS_ assay. HD PBMCs were stained with the viability dye eFluor 780 (eF780), the DNA dye Hoechst-33342, and fluorochrome conjugated mAbs against surface markers and Ki-67, as described (16). An example of flow cytometry analysis is shown. **(A)** Gating of viable single CD8 T cells in 6 steps: 1) DNA-A/-W singlets. Single cells having 2n≤ DNA content ≤4n were selected on the DNA-area (A) *versus* (*vs*) DNA-width (W) plot; 2) Time exclusion. Stable acquisition over time (seconds) was monitored on the time *vs* DNA-A plot and any events collected in case of pressure fluctuations were excluded; 3) Viable cells, no “dump”. Cells expressing CD4, CD14 and CD19, and dead cells were excluded; 4) FSC A/SSC-A “relaxed” gate. A “relaxed gate was used on the FSC-A *vs* SSC-A plot, to include highly activated and cycling lymphocytes (15); 5) CD8 T cells. CD8 T cells were gated on the CD3 *versus* CD8 plot; 6) Refined singlets. A few remaining doublets composed by one cell sitting on top of another (so called “shadow” doublets) were excluded as Ki-67int/^-^ events having > 2n DNA content (16). This gating strategy was used as a base for the subsequent gates. **(B)** The following naïve/memory subsets of CD8 T cells were identified: CD45RA^+^ CCR ^+^ Naïve, CD45RA^-^ CCR7^+^ central memory (CM), CD45RA^-^ CCR7^-^ effector memory (EM), and CD45RA^+^ CCR7^-^ (EMRA). **(C)** Cell cycle phases of each naïve/memory CD8 T cell subset were defined on DNA-A *vs* Ki67-A plot as follows: cells in G_0_ were identified as DNA 2n/Ki67^-^ (bottom left quadrant); cells in G_1_ as DNA 2n/Ki67^+^ (upper left quadrant); cells in S-G2/M (or TDS cells) as DNA>2n/Ki67^+^ (top right quadrant). Unpublished data in relation to (16).

**“**HD PBMCs were stained with the viability dye eFluor 780 (eF780), the DNA dye Hoechst-33342, and fluorochrome conjugated mAbs against surface markers and Ki-67, as described (16). An example of flow cytometry analysis is shown. **(A)** Gating of viable single CD8 T cells in 6 steps: 1) DNA-A/-W singlets. Single cells having 2n≤ DNA content ≤4n were selected on the DNA-area (A) *versus (vs)* DNA-width (W) plot; 2) Time exclusion. Stable acquisition over time (seconds) was monitored on the time *vs* DNA-A plot and any events collected in case of pressure fluctuations were excluded; 3) Viable cells, no “dump”. Cells expressing CD4, CD14 and CD19, and dead cells were excluded; 4) FSC-A/SSC-A “relaxed” gate. A “relaxed” gate was used on the FSC-A *vs* SSC-A plot, to include highly activated and cycling lymphocytes (15); 5) CD8 T cells. CD8 T cells were gated on the CD3 *versus* CD8 plot; 6) Refined singlets. A few remaining doublets composed by one cell sitting on top of another (so called “shadow” doublets) were excluded as Ki-67^int^/^-^ events having > 2n DNA content (16). This gating strategy was used as a base for the subsequent gates. **(B)** The following naïve/memory subsets of CD8 T cells were identified: CD45RA^+^ CCR7^+^ Naïve, CD45RA^-^ CCR7^+^ central memory (CM), CD45RA^-^ CCR7^-^ effector memory (EM), and CD45RA^+^ CCR7^-^ (EMRA). **(C)** Cell cycle phases of each naïve/memory CD8 T cell subset were defined on DNA-A *vs* Ki67-A plot as follows: cells in G_0_ were identified as DNA 2n/Ki67^-^ (bottom left quadrant); cells in G_1_ as DNA 2n/Ki67^+^ (upper left quadrant); cells in S-G_2_/M (or T_DS_ cells) as DNA>2n/Ki67^+^ (top right quadrant). Unpublished data in relation to (16).”

In the original article, there was also a mistake in the legend for **Supplementary Table 1** as published. The peptide- HLA-A*02 tetramer list was incorrectly formatted, there was missing information about numbers in the table (they represent average percentages); missing information about the number of mice (panel A) and number of human donors (panel B and C); and a missing citation of original references at the end. The corrected **Supplementary Material File** is linked below.

In the original article, there was also a mistake in **Figure 1** as published. **There was an incorrect y-axis label in panel A, third graph from left.** The corrected **Figure 1** appears below.

The authors apologize for these errors and state that they do not change the scientific conclusions of the article in any way. The original article has been updated.

## Publisher’s Note

All claims expressed in this article are solely those of the authors and do not necessarily represent those of their affiliated organizations, or those of the publisher, the editors and the reviewers. Any product that may be evaluated in this article, or claim that may be made by its manufacturer, is not guaranteed or endorsed by the publisher.

