# Supplementary material for: To Ki or Not to Ki: Re-Evaluating the Use and Potentials of Ki-67 for T Cell Analysis
Source: Front Immunol. 2021 Apr 9;12:653974. doi: 10.3389/fimmu.2021.653974 (PMC8062736; doi:10.3389/fimmu.2021.653974)
Supplement: Supplementary file 1 [file Table_1.pdf]

Supplemental Table 1. T_{DS} assay for immuno-monitoring

A) Vaccinated mice

	gag-specific CD8 (1 epitope) vaccinated mice		total CD8 untreated mice	
	% in G_1	% in S-G_2/M (T_{DS})	% in G_1	% in S-G_2/M (T_{DS})
day 3	22.36	4.98 (** vs day 44)	3.82	0.18
day 7	76.28 (** vs day 44)	0.61	5.05	0.20
day 44	1.56	0.02	6.67	0.17

B) T1D patients

	islet-specific CD8 (5 epitopes)		total CD8	
	% in G_1	% in S-G_2/M (T_{DS})	% in G_1	% in S-G_2/M (T_{DS})
T1D patients, T_{DS} positive (T1D $T_{DS}{}^{+}$)	15.25	1.79 (** vs HD; *** vs T1D $T_{DS}{}^{-}$)	2.14	0.11
T1D patients, T_{DS} negative (T1D $T_{DS}{}^{-}$)	2.09	0.00	1.04	0.02
Healthy donors (HD)	2.63	0.05	1.41	0.04

C) COVID-19 patients

	CD4 T_{EM}		CD8 T_{EM}		$\gamma\delta$ T cells	
	% in G_1	% in S-G_2/M (T_{DS})	% in G_1	% in S-G_2/M (T_{DS})	% in G_1	% in S-G_2/M (T_{DS})
Severe COVID-19	12.89 (***** vs HD) (** vs Mod)	1.25 (***** vs HD) (**** vs Mod)	20.78 (***** vs HD)	1.43 (***** vs HD) (* vs Mod)	9.87 (***** vs HD) (*** vs Mod)	0.33
Moderate COVID-19 (Mod)	8.08 (*** vs HD)	0.50 (** vs HD)	12.88 (**** vs HD)	1.02 (* vs HD)	7.49 (** vs HD)	0.18 (* vs HD)
Healthy Donors (HD)	3.45	0.12	2.27	0.04	1.67	0.02

Dual Ki-67/DNA staining was combined with peripheral blood T cell multi-color flow cytometric analysis, for a refined immuno-monitoring evaluation including cell cycle. **(A)** BALB/c mice were vaccinated by prime with the viral vector ChAd3 carrying the model antigen HIV-1 gag (gag), and boost with Modified Vaccine Ankara carrying gag, both administered intramuscularly in the quadriceps. Analysis of $gag_{197\text{-}205}$-specific and total CD8 T cells was performed at the indicated times after boost in vaccinated and untreated mice, respectively (15). **(B)** Peripheral Blood Mononuclear Cells (PBMCs) from Type 1 Diabetes (T1D) patients, all within 1 year from diagnosis, and healthy donors (HD) were analyzed, using a pool of the following tetramers to identify islet-specific CD8 T cells: $PPI_{15\text{-}24}$, $InsB_{10\text{-}18}$, $GAD_{114\text{-}123}$, $IGRP_{265\text{-}273}$, and IA-$2_{797\text{-}805}$ HLA-A*02 tetramers. $T_{DS}{}^{+}$ and $T_{DS}{}^{-}$ T1D patients were defined as those having > and < 0.248% T_{DS} (i.e. HD mean + 3xSD) among islet-specific CD8 T cells, respectively (16). **(C)** PBMCs were obtained from HD and hospitalized COVID-19 patients, classified according to a World Health Organization's (WHO) eight-point scale as moderate (scores 3-4) and severe (scores 5-8). Patient classification reflected peak severity. CD4 T_{EM}, CD8 T_{EM}, and $\gamma\delta$ T cells were analyzed (17). Numbers represent mean percentages of cells in G_1, and in S-G_2/M (T_{DS}) from a total of 45 mice in (A), and of 21 and 104 donors in (B) and (C), respectively. Statistical analysis was performed by Kruskal-Wallis with Dunn's multiple comparison test (A and B) (15, 16), and by a linear mixed model grouped by severity, with patient as random variable, corrected for age- and sex-dependency (C) (17). Differences were considered statistically significant when * P=.05, ** P=.01, *** P=.001, **** P=.0001, ***** P=.00001. See original references for more details (15-17).
